# Determining the Arrhenius Kinetics of Avocado Oil: Oxidative Stability under Rancimat Test Conditions

**DOI:** 10.3390/foods8070236

**Published:** 2019-06-30

**Authors:** Tugba Aktar, Eda Adal

**Affiliations:** 1 Food Engineering Department, Faculty of Engineering, Alanya Alaaddin Keykubat University, 07450 Antalya, Turkey; 2 Food Engineering Department, Faculty of Engineering, Gaziantep University, 27310 Gaziantep, Turkey

**Keywords:** avocado oil, accelerated shelf life testing, kinetic behavior, functional oil, rancimat, oil technology

## Abstract

Avocado is a highly potential functional fruit with significant health benefits which has high demand for consumption with a preferable taste. The fruit is one of the oil sources that still needs further examination on its probable kinetic behavior and oxidative stability as well as some characteristic behavior to commercialize and increase the market demand as functional oil. Hence, this study was motivated primarily for obtaining the Arrhenius kinetic information about avocado oil to evaluate the oxidative stability and provide predictive information about the shelf life by using the Rancimat method which is an accelerated shelf life test. Specifically, this research paper presents the study of the physical, physicochemical, chemical, and oxidative stability tests with the shelf life expectancy and kinetic property of avocado oil. According to the analyses, avocado oil has 210 days of predicted shelf life at 25 °C. This gives it a greater chance to be considered a good alternative to other oils as well as its antioxidant and phenolic content. According to the findings presented in this study, avocado oil has a very similar profile to olive oil and can be used as an alternative functional oil source.

## 1. Introduction

Avocado (*Persea americana Mill*) is a fruit with high-calorie content, which is considered as a functional food due to lipid fraction. It is one of the ancient fruits known have survived from the Aztec times and has been used as vegetable butter since then [1]. Avocados grow at tropical and subtropical regions and belong to the family of *Lauraceae*, which has about 150 species all with a similar lipid profile to olive oil [2]. The main reason avocados have attracted the attention of researchers and consumers is because of their functional compounds, which provide additional properties than just oil [3]. The fruit flesh consists of 30% oil. Typical avocado oil has about 75% monounsaturated fat that comprises oleic and palmitoleic acids, where 25% is saturated and polyunsaturated fatty acids [2]. The avocado oil has a characteristic flavor and a high smoke point that is above 250 °C. The oil has a green color and avocado flavor with mushroom flavor notes. This makes it worth promoting sensory aspects in the future. More importantly, the main functional component is the α-tocopherol, which is an antioxidant chemical that is usually around 79 to 190 mg/kg in avocado oil [1]. In the literature, the avocado has been investigated as fruit and source of vegetable oil, and studies are mostly focusing on the characterization of the oil, physicochemical properties, fatty acid composition, and comparative studies with olive oil [4,5,6].

To obtain the maximum functional components, the fruit needs to be carefully processed into the oil with extraction methods such the soxhlet extraction, pressing, and ultrasonic extraction [7]. Not only for avocado but also for all the vegetable–fruit oils, quality parameters are crucial for legislation as well as consumers safety. While extracting the oil from the source, heat is generally applied for conventional extraction methods. When applying heat, many desirable technological advantages occur, as well as undesirable reactions. Oils are sensitive to heat and undergo quality loss caused by chemical instability [7,8]. The most critical chemical change that represents quality loss and deterioration in the oil is lipid oxidation. This well-studied field of oil occurs due to the chain reactions that result in the formation of various oxidation products that are not suitable for consumption. The oxidation procedure is the result of heat as well as environmental conditions, such as oxygen. Effects of the lipid oxidation can be analyzed from the oxidized product; however, we can also express those effects by mathematical relationships. Most often, the cause of the oxidation is the temperature, especially while storing the oil/fat, and dependence of the temperature can be expressed best by the Arrhenius model [9]. However, a researcher should always keep in mind that this mathematical model can only be applied to a simple food system since the oxidative reactions are product dependent and often more complex [10]. To apply the best fitting mathematical relationship to oil, it is necessary to obtain information about the chemical structure, as well as manufacturing and processing conditions. Kinetic data helps us to understand the oxidation reaction to calculate the best conditions to obtain better quality with minimum deterioration. Additionally, using kinetic data, we can predict the oxidative stability changes under various temperature applications, and storage and transporting periods [7].

Oil pressing/extraction technology is facing changes in the main manufacturing processes due to the expected trend of cold pressing for better flavor, color, and higher functional nutrient content. Apart from the mentioned advantages, cold pressing leads to a lower oxidation probability. For other fruits and seeds such as olive oil, cold-pressing has become the conventional method. 

Production volume of the avocado cold press oil is 2000 tonnes/year globally [11]. Despite the increasing demand on the unsaturated functional oils, there is a limited number of studies on the characterization of the avocado oil and the effect of production technologies on the quality. The main reason for the cold pressing demand is due to easy and low-cost manufacturing as well as nutritional protection. On the other hand, non-thermal applications were found to be beneficial for the physical and chemical characteristics of the oil but disadvantaged in terms of extraction efficiency [12]. 

This paper presents innovative information about the mathematical relationships of the kinetics of avocado oil as well as including some characteristic information about the oil source. The main objective of this study was to develop mathematical models to describe the reaction rate as a function of temperature for avocado oil to explore the shelf life and storage conditions, to provide insight into the new generation functional oils and also to provide a step for future studies that are essential for avocado oil. On the other hand, antioxidant properties and some characteristic properties were determined, which might be either missing in the literature or need confirmation. 

## 2. Materials and Methods

Avocado fruits (Fuerte and Hass variety) were collected from the local market of Alanya, a town of Antalya city (Turkey) from a registered farmer. From each variety, 2 kg of fruit were purchased, which is good enough for the analysis. All chemicals were purchased from Sigma-Aldrich Chemical Company (St. Louis, MO, USA). Chemicals that used GC analysis were of chromatographic grade. Other chemicals and solvents were in analytical grade. 

### 2.1. Extraction of Avocado Oil

Avocado oil was extracted as applied previously in the literature [13]. Selected fruits were washed and deseeded. The avocados were then mashed and transferred to a stainless steel vessel (malaxer). An adequate amount of water was added, and the mixture was mixed for 60 min at 45 °C. The mixture was then centrifuged at 5000 rpm for 20 min. (Eppendorf 5810R, Hamburg, Germany). Finally, the oil layer was removed, placed into a dark brown bottle and stored at 4 °C until analysis.

### 2.2. Kinetic Data Analysis

Oxidative stability of avocado oil was determined by the rancimat method, which is considered as an accelerated determination of oxidation [14]. The rancimat method evaluates the stability by measuring the oxidation induction time, with the Rancimat apparatus (Metrohm 743, Herisau, Switzerland) which is capable of operating over a temperature range of 50 to 220 °C. During the analysis, rancimat vessels containing 3 g of oil samples were placed in an electric heating block. Effluent air containing volatile organic acids from the oil sample was collected in a measuring vessel containing distilled water (60 mL). The conductivity of water was measured automatically as oxidation proceeded. Filtered, cleaned, and dried air was allowed to bubble through the hot oil at 20 L/h. The oxidation induction time (OIT) of the oil samples were then automatically recorded at 100, 110, 120, 130, and 140 °C. OIT is the time taken until there is a sharp increase in conductivity, which is determined by the intersection of the baseline with the tangent to the conductivity curve [15]. Between each run, the glassware was rigorously cleaned to avoid any contamination that would catalyze the peroxidation. The tubes were cleaned with acetone after each run and then washed off with washing liquid and hot water. The washed tubes were rinsed with distilled water and dried in the oven. Measuring vessels, electrodes, and connecting tubes were cleaned several times with alcohol and distilled water. For each parallel test, eight samples were accommodated in the equipment and analyzed simultaneously. Kinetic parameters provide information about lipid oxidation. At moderate temperatures, due to the high concentration of oxygen dissolved in the oil, the lipid is prone to the autoxidation regardless of the oxygen pressure and oxidation rate [16,17,18]. In this study, the effect of temperature on the rate of lipid oxidation was illustrated by means of the Arrhenius equation:

$$\ln\left(k\right)=\ln A-\frac{E_{a\;}}{RT}$$

where *k* is the reaction rate constant or reciprocal oxidation induction time (OIT), *A* is the pre-exponential factor or frequency factor, *E*_a_ is the activation energy (kJ mol^−1^), *R* is the molar gas constant (8.314510 J K^−1^ mol^−1^), and *T* is the absolute temperature (K). 

The OIT of the oil determined activation energy, and frequency factors were calculated from the slopes and intercepts of the lines generated by regressing ln (*k*) vs. 1/*T* by use of the least squares linear regression, respectively. By those values and calculations, the shelf life prediction of avocado oil at 25 °C was calculated by linear regression of log OIT versus *T* in K, which adapted from the literature [7,19]. 

### 2.3. Determination of Basic Physical and Physicochemical Properties of Avocado Oil

#### 2.3.1. Density of Avocado Oil

The density of the avocado oil was measured using pychnometric methods according to the International Organization of Standardizations (“Methods for determining the density of non-cellular plastics, Part 1: Immersion method, liquid pyknometer” (ISO 1183-1:2012)). Measurements were taken of the volume of the oil displaced by a known mass of the samples at 25 °C. According to that reading, the density value of the oil samples was calculated by taking the ratio of mass to volume.

#### 2.3.2. Refractive Index

Refractive indices (optical measurements) of the oil samples were measured by using an Abbe Refractometer (ATAGO NAR 1T-LIQUID, Tokyo, Japan). A halogen lamp was used for the high bright white light source. 

#### 2.3.3. Free Fatty Acid

The free fatty acid of the avocado oil was measured as one of the physicochemical properties, and the method of measurement was used for sample titration [20]. For the measurement, diethyl ether was used, and the indicator was phenolphthalein. Neutralization was done by NaOH, and the total volume of the NaOH was measured for the percentage of free fatty acid (oleic acid) content as follows:

$$\%\;\mathrm{Free}\;\mathrm{fatty}\;\mathrm{acids}=\frac{V\times 28.2\;\times \;N}{m}$$

where *V* = volume of 0.1 N NaOH used for the sample, *N* = normality of NaOH used for titration, and *m* = mass of the sample in grams

### 2.4. Determining the Chemical Properties of Avocado Oil

#### 2.4.1. Determination of Fatty Acid Composition of Avocado Oil

The fatty acid composition of avocado oil was analyzed using Agilent GC7890A system (Agilent Technologies, Wilmington, DE, USA) gas chromatography equipped with a flame ionization detector and HP-88 capillary column (88% Cianopropylaryl; 100 m × 0.250 mm ID × 0.20 µm film thickness). The flow rate of the carrier Helium gas was set to 1 mL per minute, where detector temperature and injector temperature was 250 °C, respectively. The oven temperature was set to start with 175 °C for 10 minutes and increase 5 °C per minute up to 230 °C. Injection volume was 1 µL. These analytical conditions were used for fatty acid methyl esters (FAME) extraction with n-heptane after cold methylation with 2N KOH in methanol. Specifically, fatty acids were converted into FAME by 0.1 g of the oil sample being mixed with 2 mL n-heptane, which followed by addition of 0.2 mL (2N) methanolic KOH addition. After the phase separation GC analysis was initiated. 

The specific standard for each fatty acid type was used for the identification of the fatty acids by comparing the retention times for standards and samples. The area was expressed as percentages of the areas of the total fatty acids [21].

#### 2.4.2. Determining the Total Phenolic Content of Avocado Oil

The total phenolic contents of the avocado oil were assessed with the Folin–Ciocalteu method [22]. Total phenolic content was calculated using a standard curve of gallic acid and reported as milligram gallic acid equivalent milligram per grams of oil (mg of GAE g^−1^). Briefly, the reaction mixture contained 0.1 mL of oil extracts and 0.5 mL of the Folin–Ciocalteu reagent freshly prepared in the laboratory and 1.5 mL of 20% sodium carbonate and was completed to 10 mL volume with pure water. After 2 h of reaction at ambient temperature, the absorbance at 765 nm was measured, and the obtained value was used for the calculation of the phenolic contents in oil using gallic acid as a standard. 

#### 2.4.3. Determination of 2,2-Diphenyl-1-picrylhydrazyl (DPPH) Radical Scavenging Activity

DPPH scavenging capacity of the avocado oil was determined according to the previously reported method applied previously [23]. During the experiments, a stable 2,2-diphenyl-1-picrylhydrazyl (DPPH) radical was used, which was purchased from Sigma-Aldrich (St. Louis, MO, USA). Freshly made 100 mM DPPH-MeOH solution was mixed into the oil extract at different concentration of mg oil equivalents/mL to start the radical–antioxidant reaction. The absorbance at 517 nm was measured against a blank of pure methanol after 30 min of the reaction.

#### 2.4.4. Component Analysis of Avocado Oil

Fourier transforms infrared radiation (FTIR) is one of the trending analytical tool for edible oils [24]. FTIR is a quantitative tool that carries out component analyses. In this study, the spectral data were collected on a Perkin–Elmer Spectrum 100 spectrophotometer (Spectrum Two) (Shelton, CT, USA) fitted with a universal attenuated total reflectance (UTAR) sampling device. A drop of each oil sample was placed directly onto the Universal diamond ATR crystal, and all spectra were measured at room temperature against a background spectrum of air in the wavenumber range from 4000 to 600 cm^−1^. Between each sampling procedure, the cell was thoroughly cleaned and dried by aspirating hexane through the cell using a vacuum and its cleanliness verified spectrally. Spectra were examined using the instrument's software Spectrum 10 STD (Perkin-Elmer, Shelton, CT, USA) with peak heights and areas computed from the raw spectra. 

### 2.5. Statistical Analyses

All experiments and measurements were made in triplicate for each replicate. The coefficient of variation for quadruplicate determinations was typically less than 5%. All data were subjected to ANOVA and regression analyses using the SPSS statistical package (Version 22, SPSS Inc., Chicago, IL, USA). Duncan’s multiple range tests were applied to measure specific differences between pairs of means. General statistical values, such as mean value, correlation of determinations (*R*^2^), *p*-values, and standard deviation, were calculated. Additionally, the analysis made on the avocado oil were grouped as Kinetic data analysis (Rancimat test), physical and physicochemical analyses (density, refractive index, free fatty acid content), chemical analyses (accelerated oxidation, fatty acid composition, total phenolic content, DPPH radical scavenging), and component analyses (FTIR). Each experiment was done in triplicate, and the mean data were calculated

## 3. Results and Discussion

The main aim of this study was to characterize and investigate the kinetic analysis of avocado oil. The rancimat method was used as a reflection of the expected shelf life determination as well as determining the kinetic behavior of the avocado oil. Rancimat measurements for the oxidation induction period (OIT, time in hours) were done isothermally at five different temperatures (100 °C, 110 °C, 120 °C, 130 °C, and 140 °C) and those values were selected according to the study done previously [25]. OIT of the avocado oil can be seen in Table 1. Results illustrate that OIT values are decreasing by the increasing temperature. This result has been previously validated [26] According to the experiments and calculation, a linear regression model was obtained, which was used for calculating the expected shelf life of the avocado oil.

As mentioned earlier, the *k* value (reaction rate constant) was determined by the reciprocal OIT for each temperature and represents lipid oxidation of avocado oil, which can be seen for different temperature operations in Table 1. By using the *k* value rate of lipid oxidation as a function of temperature, a direct relationship can be observed. 

**Figure 1:**
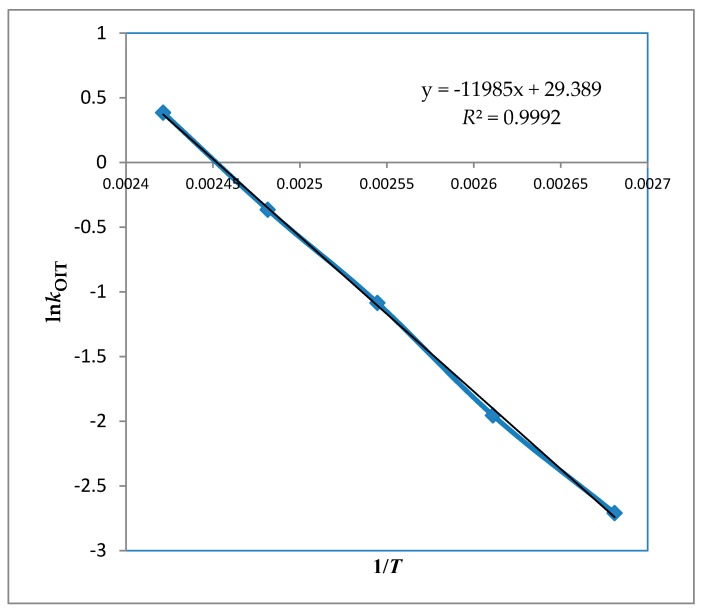
ln (*k*) vs. 1/*T* which illustrates the activation energy and pre-exponential factor where the slope is used for the calculation of *E*_a_ while the intercept is used for the calculation of *A* (frequency factor).

As shown in Figure 1, ln (*k*) vs. 1/*T* was obtained from the measurements. Those values were used to obtain activation energy and pre-exponential factor. The slope was used to calculate *E*_a_ while intercept used for *A* (frequency factor) as explained in (*R*^2^ > 0.99). More specifically, *k* and *T* values have a semi-logarithmic relationship with a linear dependency with good correlation of determination (*R*^2^ > 0.99). Duncan’s multiple range tests were calculated for OIT assay and statistically significant (*p* < 0.05) difference was found at all temperatures. The results indicate that the reaction rate constant increase with increasing temperature, which means that the oil oxidation is faster at higher temperatures as authors expected. Noteworthy, the kinetic rate constant prediction, especially at lower temperatures, has some limitations which cause some uncertainties and errors. Those limitations are linked with the test. Specifically, the oil follows different pathways of lipid oxidation at lower and higher temperatures depending on the metal ions and antioxidants activity. On the other hand, the degree of oxygen solubility is different in varying temperatures, where literature reporting to see an increase of 25% for each 10 °C. 

Calculated Arrhenius activation energies (99.6 ± 2 kJ·mol^−1^) and pre-exponential factor (5.8 × 10^12^ h^−1^) are listed in Table 2. According to the literature, the stability of avocado oil was similar to that of olive oil because of similar lipid profile and stability results, which means our results are in accordance with literature findings [11]. Another supporting data for the similarity of avocado oil and olive oil was investigated on different kinds of olive oils under rancimat conditions and illustrated that the activation energies for the oxidation of the olive oils were between 97.7 kJ·mol^−1^ and 101.9 kJ·mol^−1^, which is very similar to the findings in this study [25]. A previous investigation showed that the kinetics of different type of vegetable oils, as well as *E*_a_ values of selected vegetable oils, was found to be between 79 mol^−1^ and 104 kJ·mol^−1^ which have a different fatty acid profile [7]. Alternative oils, such as vegetable oils and olive oil, were found to have similar activation energies. The oils which have lower activation energy are expected to require a higher temperature to induce a certain change in the rate of oxidation. Therefore according to our findings, avocado oil is stable compared to other oil sources as well as olive oil. 

**Figure 2:**
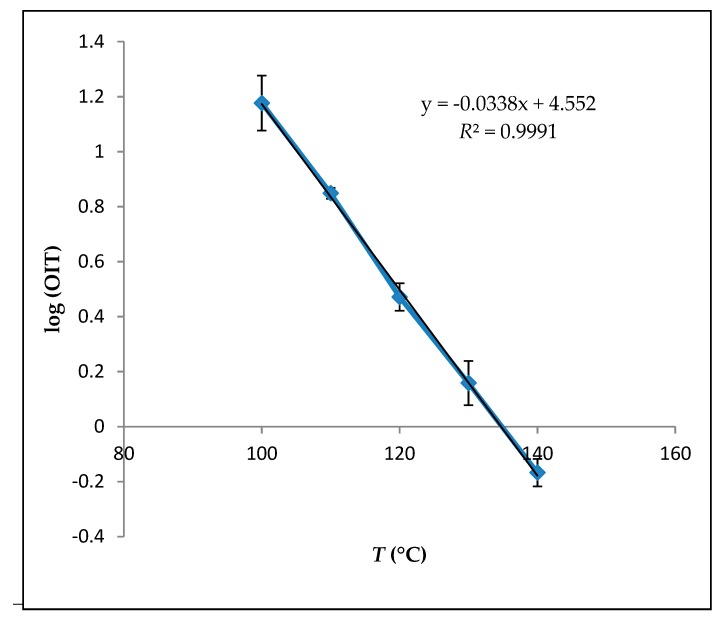
The linear relationship between the natural logarithm of the oxidation induction time (OIT) and the temperature.

Kinetic properties calculations were based on the measurements done by the Rancimat method to investigate the expected shelf life of avocado oil. A linear relationship between the natural logarithm of the OIT and the temperature was used to calculate the predicted shelf life of avocado oil at 25 °C, as shown in Figure 2. 

It can be observed that avocado oil obeys the Arrhenius relationship over the temperature range of 100 °C to 140 °C (*R*^2^ > 0.99). The data calculated from this relationship are shown in Table 3. According to those calculations, the temperature coefficient of avocado oil was −3.4 × 10^−2^ °C^−1^ (logOIT = a(*T*) + b). Kinetic behavior of the avocado oil has not been studied previously according to our literature knowledge. Therefore, the kinetic behavior of the avocado oil is still missing, and this study will trigger the further research ideas of the avocado oil. Previously other oil sources, such as soybean oil, were tested, and the temperature coefficient was found as −3.12 × 10^−2^ °C^−1^ [27]. Another study on the vegetable oil temperature coefficient was calculated as −2.78 × 10^−2^ and −3.15 × 10^−2^ °C^−1^ (mean value −3.01 × 10^−2^ °C^−1^) [28]. Even though those findings are not necessarily comparable with the avocado oil, they seem to be fitting with our findings and calculations as a general profile. 

Noteworthy, rancimat tests of the ambient storage were declared to lead either over prediction or under prediction of the actual shelf life depending on the type of oil [27]. Additionally, the lipid oxidation at low and high temperatures may go through different reaction pathways, depending on the reactivity of metal ions and antioxidants at different temperatures [19]. Therefore, the rancimat method can be claimed as a remarkable kind of a test as a fast shelf life prediction, but it would be suitable to compare fast and regular shelf life tests in future researches. 

According to our calculations, avocado oil has 210 days of shelf life according to the model. The usual shelf life of the vegetable oils was presented to be between 12 and 15 months at 25 °C when good manufacturing and good storing conditions are followed [26]. Additionally, it is also necessary to highlight that once the oxygen interaction initiates, shelf life starts to decrease. However, the addition of the alternative antioxidants, minor protective/antioxidative components may increase the shelf life, which is still in need of further investigation to obtain the best component and best practice.

Results of the physical and physicochemical properties, specifically density, refractive index, and free fatty acid measurements are shown in Table 4. The density of the avocado oil was 0.91 g/mL (±0.0001). Meanwhile, the refractive index value was 1.4680 (±0.0002). For the density and refractive index values, literature has reported similar results [6,29]. Those properties are similar to those of olive oil. In the literature, virgin olive oil was presented to have 0.8639 g/mL density while the refractive index was between 1.480 and 1.465 according to varying wavelength values of experiments [30].

The free fatty acid value was found to be 1.065% (±0.040) oleic acid. An earlier study illustrated that free fatty acid value of the virgin olive oil and extra virgin olive oil was measured as 0.26% and 0.36%, respectively [31]. It can be seen that the acid value of the avocado oil is higher than the olive oil. Those values imply that avocado oil has similar characteristic properties to olive oil, which has also been supported by previous researches [11,32,33]. Noteworthy, oil processing technique is critical and varies the free fatty acid values [12]. Therefore, the effect of different oil pressing techniques on the free fatty acid value can be a further research question.

Results of fatty acid compositions of avocado oil were illustrated in Table 5. The profile shows that obtained fatty acids are mostly unsaturated except for the palmitic acid. Specifically, avocado oil contains (in descending order) oleic acid (18:1 ω-9), palmitic acid (16:0), linoleic acid (18:3 ω-3), and palmitoleic acid (16:1). The high content of unsaturated fatty acid gives a unique property to avocado oil with delivering functionality to the product. Obtained fatty acid profile agrees with previous reports [34,35,36,37]. However, the differences in the cultivars of the fruit might change the concentration of the fatty acids presented, but it is likely to have a similar order of the fatty acid concentration [38]. 

Due to the potential impact on human health and critical importance for the daily diet, the total phenolic content of the avocado oil was measured. The daily intake of the total phenolic content was estimated to be sourced by the most common 34 fruits and vegetables where avocado was one of them [39]. Therefore, in this perspective, avocado is one of our phenolic source where it worth doing examination about its total phenolic content and the methods to increase the usage of it. In our study, the total phenolic content was found as 25.73 (±2.1) mg GAE per g of oil (Table 6). The previous study done by [39] presented that avocado fruit contains 33.62 mg GAE per g of oil. With a similar approach [40] different cultivars of avocados found that they between 6 and 49 mg GAE per g of oil. The total phenolic content can get affected by the maturity state of the avocado, and the more mature fruit was found to have higher phenolic content [41]. On the other hand, olive oil has shown similar results to those of avocado oil, and this finding supports the idea of avocado oil being an alternative oil [42,43].

Total antioxidant capacity varies from one kind of fruit/seed to another. Additionally, the total antioxidant capacity is easily affected by the processing methods as well as the different parts of the fruits [43]. It should be highlighted that for functional oil (which is likely to contain antioxidants) resistance to oxidation stress is critical. There are several methods for measuring the total antioxidant capacity, and these methods generate different radical/target groups [44,45]. In this study, the DPPH method was used to evaluate the antioxidant capacity, which is also a very common method for the assessment of antioxidants [46,47]. According to the analysis done, the antioxidant capacity of avocado oil was found to be 32.4 mg/mL (±1.3) as IC_50_ value, required to lower the initial DPPH concentration by 50% (Table 6). A previous research illustrated the antioxidant capacities of the extra virgin olive oil, olive oil, corn oil, sunflower oil, and soybean oil as 15 mg/mL, 22 mg/mL, 52 mg/mL, 48 mg/mL, 45 mg/mL, respectively [47]. The results showed that the radical scavenging capacity of avocado oil is higher than those of corn, sunflower, and soybean oil.

**Figure 3:**
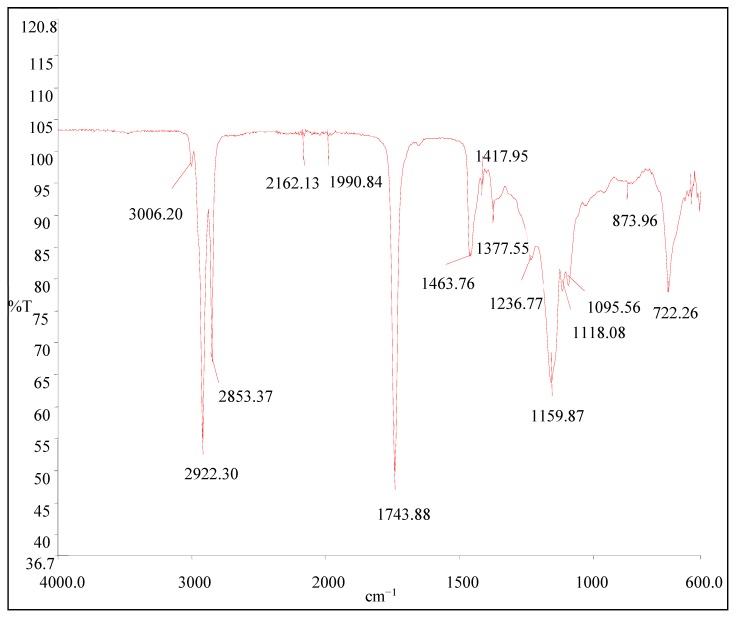
FTIR spectra of avocado oil in the region of 4000 to 600 cm^−1^.

FTIR results are shown in Figure 3. The spectral regions were chosen for developing the regressions with including the fingerprint regions were selected according to the previous researcher's observations [48]. The spectra in the FTIR region have well-resolved bands that can be assigned to presented functional groups of the avocado oil. The spectra are dominated by some peaks at 3006, 2922, 2853, 2162, 1991, 1744, 1464, 1418, 1378, 1237, 1160, 1118, 1096, 874, and 722 cm^−1^. According to that, the absorbance results for the region of 3006 to 2853 cm^−1^ are due to the bands of CH_2_ stretching vibrations, asymmetric and symmetric, respectively. The high value of the frequency of this band indicates its richness in polyunsaturated acyl groups. According to the literature, only linseed oil shows a band frequency as high as this; for example, olive oil shows values near 3005.4, rapeseed oil near 3007.5, and corn oil near 3008.8 cm^−1^ [49]. So, the frequency value of these bands in avocado oil is similar to those of olive oil. The major peak at 1744 cm^−1^ arises from C=O stretching vibrations of aldehydes and ketones where the peaks at 1464, 1418, and 1378 cm^−1^ arise from CH_2_ and CH_3_ scissoring vibration of ethers. Meanwhile, 1237, 1160, 1118, and 1096 cm^−1^ peaks are associated with C–O stretching vibration. The last peak at 722 cm^−1^ associates with the CH_2_ rocking mode. Those observations are supported by the results of the other researchers performed with oils [48,50].

## 4. Conclusions

According to the analyses and literature comparison of the characteristic values of avocado oil, it can be mentioned that avocado oil has similar characteristic behavior to the olive oil which makes it a high potent functional oil. On the other hand, the shelf life was determined to be a little above 200 days. However, 200 days is still a feasible duration for storage time for a functional oil. Kinetic behavior was determined by the temperature coefficient, and it was found as −3.4 × 10^−2^ °C^−1^. This data has not been studied earlier and according to other studies for the temperature coefficient value was found to be −3.12 × 10^−2^ °C^−1^, −2.78 × 10^−2^ and −3.15 × 10^−2^ °C^−1^ for soybean oil and other vegetable oils, relatively [19,27,28]. Those findings are similar to those of avocado oil, which could mean that avocado oil can be an alternative oil source.

In conclusion, cold pressed avocado oil which has already been previously referred as a healthy alternative oil and according to the illustrated results and calculations; it can be an alternative not only to other edible oils but also to olive oil.

## Figures and Tables

**Table 1:** Oxidation induction time (OIT) values and reaction rate constants (*k*_OIT_ = 1/OIT) at five different temperatures T for avocado oil.

T (°C)	OIT (h) *	*k*_OIT_ (×10^−3^ h^−1^)
100	15.02 ± 2.03 ^a^	66.6
110	7.06 ± 3.15 ^b^	141.6
120	2.96 ± 0.25 ^c^	337.8
130	1.44 ± 0.03 ^d^	694.4
140	0.68 ± 0.15 ^e^	1470.6

* Values printed in one column, with the different letters (a–e) in superscript are statistically different at the *p* < 0.05 level, 95% confidence limit, according to Duncan’s Multiple Range Test. OIT, oxidation induction time.

**Table 2:** Estimated Arrhenius parameters for avocado oil oxidation on isothermal conditions (ln*k*_OIT_ vs. 1/*T*).

Parameters	Avocado Oil
b (intercept)	29.4 ± 0.83
a (slope)	−11985 ± 311
*R* ^2^	0.99
*E*_a_ (kJ/mol)	99.6 ± 2
*A*_OIT_ (×10^12^ h^−1^)	5.8

Relationship between lnk_OIT_ vs. 1/*T* gives a linear relationship as y = −11985x + 29.389 where coefficient of 1/*T* is represented with letter a and the additive constant of the relation is shown with letter b. Meanwhile; *R*^2^ represents regression coefficient, E_a_ represents calculated Arrhenius activation energy, and A_OIT_ represents the pre-exponential factor.

**Table 3:** Calculated results of the linear relationship between the natural logarithm of the OIT assessed by the Rancimat test and the temperature for the treatment combinations in.

Parameters	Avocado Oil
a (slope)	−0.034 ± 0.4
b (intercept)	13.8 ± 1
*R* ^2^	0.99
OIT_25 °C_ (day)	211 ± 10

**Table 4:** Physical and physicochemical properties of the avocado oil.

Measured Property	Value
Density (g/mL)	0.91 ± 0.001
Refractive index	1.4680 ± 0.0002
Free fatty acid (% oleic acid)	1.065 ± 0.040

**Table 5:** Fatty acid profile of avocado oil.

Fatty Acids	%
C14:0 (Myristic acid)	Not detected
C16:0 (Palmitic acid)	18.29 ± 0.2
C16:1 (Palmitoleic acid)	8.36 ± 0.05
C18:0 (Stearic acid)	0.69 ± 0.01
C18:1 (Oleic acid)	54.33 ± 0.4
C18:2n-6 (Linoleic acid)	11.54 ± 0.2
C18:3n-3 (Linolenic acid)	0.78 ± 0.02

**Table 6:** Total phenolic content and antioxidant activity of avocado oil.

Measured Property	Value
Total phenolic content (mg GAE/g oil)	25.73 ± 2.1
DPPH (IC_50_, mg/mL)	32.4 ± 1.3
